# Long-term outcomes of Baerveldt glaucoma implant surgery in Japanese patients

**DOI:** 10.1038/s41598-023-41673-6

**Published:** 2023-08-31

**Authors:** Kentaro Iwasaki, Ryohei Komori, Shogo Arimura, Yusuke Orii, Yoshihiro Takamura, Masaru Inatani

**Affiliations:** Department of Ophthalmology, Faculty of Medical Sciences, University of Fukui, 23-3 Shimoaizuki, Matsuoka, Eiheiji, Yoshida, Fukui 910-1193 Japan

**Keywords:** Medical research, Risk factors

## Abstract

This study evaluated the long-term surgical outcomes of Baerveldt glaucoma implant (BGI) surgery in patients with refractory glaucoma (204 eyes/204 patients). Surgical failure was defined by: < 20% reduction in preoperative intraocular pressure (IOP), or criterion A (IOP > 21 mmHg), criterion B (IOP > 17 mmHg), or criterion C (IOP > 14 mmHg). Reoperation, loss of light perception vision, or hypotony also denoted failure. The probability of success at 5 years postoperatively using criteria A, B, and C was 72.4%, 49.7%, and 24.4%, respectively. The mean IOP decreased significantly from 32.7 ± 9.7 mmHg preoperatively to 13.1 ± 3.9 mmHg at 5 years; the mean number of glaucoma medications also decreased from 3.7 ± 1.2 to 1.8 ± 1.9 (both P < 0.01). The number of previous intraocular surgeries was significantly associated with failure in the multivariable analysis for criterion B (hazard ratio 1.30; P < 0.01) and criterion C (hazard ratio 1.19; P = 0.031). Early and late postoperative complications occurred in 82 (40.2%) and 28 (13.7%) eyes, respectively. Postoperative interventions were performed in 44 eyes (21.6%). BGI surgery resulted in significant long-term decreases in IOP and the number of glaucoma medications. BGI surgery is effective for refractory glaucoma. However, postoperative interventions due to complications are required in numerous cases.

## Introduction

Tube-shunt surgery using glaucoma drainage implants has become increasingly popular for the treatment of refractory glaucoma worldwide^1^. Baerveldt glaucoma implant (BGI) surgery is considered one of the most effective glaucoma filtration surgeries. Previous studies suggested the effectiveness of BGI. The Tube Versus Trabeculectomy (TVT) study found that tube-shunt surgery using BGI with a 350-mm^2^ endplate was associated with a higher success than trabeculectomy^2^. Previous reports comparing Ahmed glaucoma valve (AGV) and BGI surgical outcomes showed that BGI surgery offered better long-term control of intraocular pressure (IOP) than AGV surgery^3–5^. Since the approval of BGIs and AGVs for use in Japan (in 2012 and 2014, respectively), tube-shunt surgery has been widely performed^6^. This type of surgery is often performed in eyes who have undergone one or more intraocular surgeries, particularly trabeculectomy. Although more than a decade has passed since the use of BGI was approved in Japan, there are no studies with large sample sizes evaluating long-term surgical outcomes. The purpose of the present study was to evaluate the long-term surgical outcome of BGI surgery in Japanese patients. We analyzed retrospective data of patients treated with BGI surgery at Fukui University Hospital (Fukui, Japan). The results may be useful for glaucoma surgeons in the selection of surgical approach.

## Methods

### Patient selection

This retrospective clinical cohort study was approved by the institutional review board of Fukui University Hospital. The protocol adhered to the tenets of the Declaration of Helsinki. All patients provided written informed consent to undergo this surgery. However, the requirement for informed consent for this study was waived owing to the retrospective nature of the investigation.

This study evaluated the surgical outcome after BGI with a 350-mm^2^ endplate (BG101-350 or BG102-350; Abbott Medical Optics, Abbott Park, IL, USA). Patients treated with BGI between April 1, 2012 and December 31, 2021 at Fukui University Hospital were recruited. The inclusion criteria were age ≥ 20 years and diagnosis of refractory glaucoma, such as secondary glaucoma, neovascular glaucoma (NVG), or glaucoma involving a conjunctival scar after previous ocular surgery. The exclusion criteria were eyes without light perception vision, eyes that had undergone previous tube-shunt surgery (BGI or AGV), or eyes that had not been followed up for more than 3 months after surgery. If both eyes in the same patient satisfied the inclusion criteria, the eye that was treated first was investigated.

### Surgical procedures

All tube-shunt surgeries were performed using a BG101-350 or BG102-350 endplate. The silicone tube was completely occluded with an 8-0 absorbable vicryl suture (Coated VICRYL; Ethicon, Somerville, OH, USA) to minimize the risk of early postoperative hypotony. A fornix-based conjunctival flap was created after administration of subconjunctival and sub-Tenon’s xylocaine anesthesia. The silicone plate was preferentially placed in the superotemporal scleral quadrant. If the superotemporal quadrant had intensive surgical conjunctival scarring, the plate was placed in one of the other quadrants. The endplate was placed under the rectus muscles and fixed on the scleral surface approximately 10 mm from the corneal limbus. A scleral tunnel was created with a 23-gauge needle to insert the tube into the anterior chamber or the ciliary sulcus. Tube insertion into the vitreous cavity through the pars plana was performed in eyes that had undergone vitrectomy. Regarding the procedure for tube insertion into the vitreous cavity, a 20-gauge microvitreoretinal-lance (MVR-lance) or 24-gauge MVR-lance were used to insert the tube with the Hoffmann elbow (BG102-350) or straight tube (BG101-350) into the vitreous space, respectively. Sherwood slits were created in the tube with the needle of the 9-0 nylon sutures to reduce the frequency of early postoperative IOP elevation. The tube was covered with a half-thickness rectangular scleral flap or a scleral patch graft supplied by the eye bank. The scleral patch and conjunctival flap were sutured using 9-0 nylon and 8-0 absorbable vicryl sutures.

All patients received similar postoperative topical medication, with 1.5% levofloxacin or 0.5% moxifloxacin thrice daily for 3 weeks, and 0.1% betamethasone sodium phosphate thrice daily for 6 months.

### Data collection

Patient data were collected, including age, sex, best corrected visual acuity, type of glaucoma, preoperative IOP, postoperative IOP, number of glaucoma medications received, previous intraocular surgeries, surgery conditions, postoperative complications, and postoperative interventions. A logarithm of the reciprocal of the decimal best corrected visual acuity was used to approximate the logarithm of the minimal angle of resolution (logMAR). Eyes without form vision were classified into one of four low-vision categories, which were assigned decimal equivalents as follows: counting fingers, 0.00500; hand motions, 0.00250; light perception, 0.00125; and no light perception, 0.00010. Oral carbonic anhydrase inhibitors were counted as one glaucoma medication.

### Primary outcome measure

Surgical success or failure was defined according to three IOP criteria. The criteria for failure were the following IOP levels, with or without glaucoma medication, at ≥ 3 months after surgery: < 20% reduction in preoperative IOP, or criterion A, B, or C, (IOP > 21, > 17, or > 14 mmHg, respectively) on two consecutive follow-up visits. IOP measurements, performed at follow-up visits every within 6 months, were used to determine surgical success. In addition, in cases that required reoperation for glaucoma (including complications that required removal of the BGI) or those that developed loss of light perception vision or had hypotony ≤ 5 mmHg, surgical failure was declared for all criteria. In cases that did not meet these failure criteria, surgery was considered successful. The prognostic factors for surgical failure were also analyzed.

### Secondary outcome measures

Secondary outcome measures included IOP, the number of medications received, early or late postoperative complications, visual acuity, and postoperative interventions.

### Statistical analysis

For continuous variables, the Mann–Whitney *U* test was used to compare two groups. The probability of success was analyzed using Kaplan–Meier survival curves. Multivariable analysis was performed to determine the prognostic factors for failure of surgery using Cox proportional hazards regression models. In the multivariable analysis, we selected important prognostic factors that may be associated with surgical failure based on previous reports and clinical experience. P values < 0.05 denote statistically significant differences. Data were analyzed with the JMP statistical package, version 11.2.0 (SAS Institute, Inc. Cary, NC, USA).

## Results

### Patient characteristics

Data of 204 eyes of 204 patients were analyzed. Table 1 summarizes the preoperative characteristics of the patients. All patients were Japanese. The mean follow-up duration for all eyes was 49.0 ± 33.3 months. Combined cataract surgery was performed in 28 eyes (13.7%). Combined vitrectomy was not performed in any of the eyes.

**Table 1:** Preoperative characteristics.

Characteristic	Total (n = 204)
Age (years), mean ± SD	68.6 ± 12.8
Sex, n (%)	
Male	124 (60.8)
Female	80 (39.2)
Visual acuity (logMAR), mean ± SD	0.89 ± 0.83
IOP (mmHg), mean ± SD	32.7 ± 9.7
Glaucoma medications, n, mean ± SD	3.7 ± 1.2
Previous intraocular surgeries, n, mean ± SD	2.1 ± 1.1
Previous trabeculotomy, n (%)	10 (4.9)
Previous filtration surgery, n (%)	89 (43.6)
Previous pars plana vitrectomy, n (%)	71 (34.8)
Type of glaucoma, n (%)	
Primary open-angle glaucoma	49 (24.0)
Primary angle-closure glaucoma	9 (4.4)
Exfoliation glaucoma	55 (27.0)
Neovascular glaucoma	51 (25.0)
Other secondary glaucoma	40 (19.6)
Lens status, n (%)	
Phakia	4 (2.0)
Pseudophakia	166 (81.4)
Aphakia	6 (2.9)
Phakia combined with cataract surgery	28 (13.7)
Type of implant, n (%)	
BG 101–350	159 (77.9)
BG 102–350	45 (22.1)
Tube insertion position, n (%)	
Anterior chamber	121 (59.3)
Ciliary sulcus	32 (15.7)
Pars plana	51 (25.0)
Endplate position, n (%)	
Superotemporal	178 (87.3)
Inferotemporal	9 (4.4)
Superonasal	16 (7.8)
Inferonasal	1 (0.5)

*IOP* intraocular pressure, *logMAR* logarithm of minimum angle of resolution, *SD* standard deviation.

### Primary outcome measure

**Figure 1:**
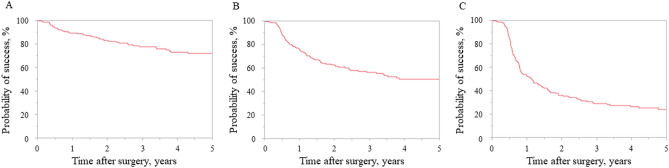
Surgical outcomes based on the Kaplan–Meier survival curves. (**a**) Criterion A: intraocular pressure (IOP) > 21 mmHg, < 20% reduction in preoperative IOP, reoperation for glaucoma, loss of light perception vision, or hypotony ≤ 5 mmHg. (**b**) Criterion B: IOP > 17 mmHg, < 20% reduction in preoperative IOP, reoperation for glaucoma, loss of light perception vision, or hypotony ≤ 5 mmHg. (**c**) Criterion C: IOP > 14 mmHg, < 20% reduction in preoperative IOP, reoperation for glaucoma, loss of light perception vision, or hypotony ≤ 5 mmHg.

Figure 1 shows the Kaplan–Meier survival curves comparing surgical outcomes according to failure criteria A, B, and C. For criterion A, the probabilities of success at 1, 3, and 5 years after surgery were 89.8%, 78.1%, and 72.4%, respectively. For criterion B, these rates were 75.3%, 56.8%, and 49.7%, respectively. For criterion C, these rates were 52.3%, 29.4%, and 24.4%, respectively.

Table 2 shows the reasons for classification as treatment failure for criterion A. A total of 51 eyes (25.0%) were classified as failures. The most common reason for failure was inadequate reduction in IOP (28 eyes, 13.7%; IOP > 21 mmHg or < 20% reduction in preoperative IOP on two consecutive follow-up visits after 3 months). Six of 28 eyes that failed because of inadequate IOP reduction subsequently underwent reoperation for glaucoma. A total of 11 eyes required additional glaucoma surgery in this study. Ten eyes were treated with micropulse transscleral cyclophotocoagulation, and one eye was treated with transscleral cyclophotocoagulation.

**Table 2:** Reasons for treatment failure.

	Total^a^ (n = 204)
Inadequate IOP reduction^b,c^	28 (13.7)
Reoperation for glaucoma	5 (2.4)
Hypotony^d^	10 (4.9)
Removal of tube shunt	4 (2.0)
Loss of light perception	4 (2.0)

*IOP* intraocular pressure.

^a^Data are presented as the number of eyes (percentage).

^b^IOP > 21 mmHg or < 20% reduction in preoperative IOP on two consecutive follow-up visits after 3 months.

^c^Some patients underwent reoperation for glaucoma subsequent to failure due to inadequate IOP reduction.

^d^IOP ≤ 5 mmHg on two consecutive follow-up visits after 3 months.

### Multivariable analysis to determine prognostic factors of surgical failure

The characteristics, including age, type of glaucoma, preoperative IOP, the number of preoperative glaucoma medications, condition of combined surgery, and the number of previous intraocular surgeries were evaluated as possible determinants of surgical failure. Table 3 shows the analyses using the multivariable Cox proportional hazards regression models. Younger age was a significant prognostic factor of surgical failure in criterion B (hazard ratio [HR] 0.98; P = 0.039). Higher preoperative IOP was associated with poorer surgical outcomes for criterion C (HR 1.02; P = 0.040). The number of previous intraocular surgeries was linked to an increased risk for criterion B (HR 1.30; P < 0.01) and criterion C (HR 1.19; P = 0.031). There was no prognostic factor identified for criterion A.

**Table 3:** Multivariable analysis to identify prognostic risk factors for failure using Cox proportional hazards regression models.

	Criterion					
	A		B		C	
	HR (95% CI)	P value	HR (95% CI)	P value	HR (95% CI)	P value
Age per year	0.99 (0.97–1.02)	0.55	0.98 (0.97–1.00)	0.039	0.99 (0.98–1.00)	0.070
Type of glaucoma (NVG/other)	1.06 (0.55–1.95)	0.87	0.88 (0.55–1.45)	0.70	0.96 (0.65–1.40)	0.84
Preoperative IOP per mmHg	1.02 (0.99–1.05)	0.10	1.02 (0.99–1.04)	0.051	1.02 (1.00–1.04)	0.040
Preoperative glaucoma medication per each	0.97 (0.76–1.26)	0.81	1.03 (0.86–1.25)	0.78	1.03 (0.90–1.19)	0.67
Combined surgery (alone/combined cataract surgery)	0.99 (0.43–2.67)	0.98	0.91 (0.50–1.81)	0.78	0.91 (0.56–1.56)	0.73
Number of previous intraocular surgeries per each	1.11 (0.86–1.40)	0.41	1.30 (1.08–1.52)	< 0.01	1.19 (1.02–1.38)	0.031

*CI* confidence interval, *HR* hazard ratio, *IOP* intraocular pressure, *NVG* neovascular glaucoma.

Supplement Table 1S shows another multivariable analysis that includes previous filtration surgery as possible determinants of surgical failure instead of the number of previous intraocular surgeries. Younger age was a significant prognostic factor of surgical failure in criterion B (hazard ratio [HR] 0.98; P < 0.01) and criterion C (HR 0.98; P = 0.022). Higher preoperative IOP was associated with poorer surgical outcomes for criterion B (HR 1.03; P = 0.024) and criterion C (HR 1.02; P = 0.016). The previous filtration surgery increased the risk for criterion B (HR 1.61; P = 0.045) and criterion C (HR 1.49; P = 0.034).

### Secondary outcome measures

Table 4 shows IOP values and the number of glaucoma medications received at various time points during follow-up. Eyes that underwent additional glaucoma surgery were excluded from the analysis after the reoperation. The mean IOP decreased significantly from 32.7 ± 9.7 mmHg preoperatively to 13.1 ± 3.9 mmHg at 5 years; the mean number of glaucoma medications also decreased from 3.7 ± 1.2 to 1.8 ± 1.9 (both P < 0.01).

**Table 4:** Intraocular pressure and glaucoma medications at preoperative and follow-up visits.

	IOP (mmHg)	Number of medications	Number of eyes
Preoperative	32.7 ± 9.7	3.7 ± 1.2	204
1 month	19.6 ± 8.3	1.6 ± 1.9	204
3 months	15.8 ± 6.8	1.5 ± 1.8	201
6 months	14.3 ± 4.9	1.5 ± 1.7	193
1 year	14.0 ± 4.8	1.6 ± 1.8	180
2 years	13.8 ± 3.7	1.8 ± 1.8	154
3 years	13.9 ± 4.1	1.8 ± 1.8	113
4 years	13.8 ± 4.1	1.8 ± 1.9	92
5 years	13.1 ± 3.9	1.8 ± 1.9	72

Data are shown as the mean ± standard deviation.

*IOP* intraocular pressure.

Table 5 shows postoperative complications. A total of 119 early postoperative complications that occurred within 3 months after surgery were observed in 82 eyes (40.2%). The most common early postoperative complication was hyphema in 49 eyes (24.0%). In almost all cases (48 eyes), hyphema resolved spontaneously without intervention. One eye required intravitreal injection of anti-vascular endothelial growth factor (anti-VEGF).

**Table 5:** Postoperative complications.

Total^a^ (n = 204)	Early^b^	Late^c^
Hyphema	49 (24.0)	0 (0.0)
Shallow or flat anterior chamber	20 (9.8)	1 (0.5)
Wound dehiscence	1 (0.5)	0 (0.0)
Choroidal detachment	25 (12.3)	0 (0.0)
Vitreous hemorrhage	18 (8.8)	4 (2.0)
Retinal detachment	1 (0.5)	0 (0.0)
IOL dislocation	1 (0.5)	0 (0.0)
Tube obstruction	0 (0.0)	1 (0.5)
Tube erosion	0 (0.0)	2 (1.0)
Removal of tube shunt	3 (1.5)	5 (2.5)
Malignant glaucoma	1 (0.5)	1 (0.5)
Endophthalmitis	0 (0.0)	1 (0.5)
Bullous keratopathy	0 (0.0)	10 (4.9)
Persistent diplopia	0 (0.0)	4 (2.0)
Corneal infection	0 (0.0)	2 (1.0)
Choroidal hemorrhage	0 (0.0)	0 (0.0)
Total number of eyes with postoperative complications^*d*^	82 (40.2)	28 (13.7)

*IOL* intraocular lens.

^a^Data are presented as the number of eyes (percentage).

^b^Early postoperative complications that occurred within 3 months after surgery.

^c^Late postoperative complications that occurred more than 3 months after surgery.

^d^Some patients had more than one complication.

A total of 31 late postoperative complications that occurred more than 3 months after surgery were observed in 28 eyes (13.7%). The most common late postoperative complication was bullous keratopathy in 10 eyes (4.9%) (i.e., seven with exfoliation glaucoma and anterior chamber tube insertion, and three with uveitic secondary glaucoma and anterior chamber tube insertion). Endophthalmitis occurred in one eye (0.5%), in which the endplate was placed at the inferior quadrant. Tube erosion occurred in two eyes (1.0%). One primary open-angle glaucoma patient who had undergone BG 101–350 in the inferonasal position encountered tube erosion at 2 years and 8 months after surgery. The patient developed endophthalmitis due to the tube erosion, and the tube was covered with a preserved scleral patch graft. At 2 years and 7 months after tube revision, tube erosion was recurred, and the tube was covered with a preserved corneal patch graft. Tube erosion has not recurred since. Another NVG patient who had received BG 102–350 in the superotemporal experienced tube erosion at 6 years after surgery, and the tube was covered with a preserved scleral patch graft. At 1 year and 6 months after tube revision, tube erosion was recurred, and the implant was removed instead of revision at the patient’s request.

Postoperative interventions are listed in Table 6. A total of 51 interventions were performed in 44 eyes (21.6%). The most common postoperative intervention was anterior chamber reformation in 16 eyes (7.8%). Anterior chamber reformation was performed using viscoelastic material when the shallow or flat anterior chamber occurred postoperatively. Intravitreal injection of anti-VEGF was required to reduce the hyphema or vitreous hemorrhage in eyes with NVG or to treat the macular edema due to diabetic retinopathy or retinal vein occlusion. Intravitreal injection of triamcinolone acetonide was required to treat the macular edema caused by diabetic retinopathy. Anterior vitrectomy was performed to treat the malignant glaucoma or release the tube obstruction by the vitreous. Pars plana vitrectomy was required to treat the vitreous hemorrhage, malignant glaucoma, retinal detachment, intraocular lens dislocation, endophthalmitis, epiretinal membrane, or vitreous opacity. All cases with preoperative phakia (four eyes) underwent cataract surgery within 2 years after surgery.

**Table 6:** Postoperative interventions.

	Total^a^ (n = 204)
Anterior chamber reformation	16 (7.8)
Intravitreal injection of anti-VEGF	11 (5.4)
Intravitreal injection of triamcinolone acetonide	2 (1.0)
Additional suture of wound	1 (0.5)
Anterior vitrectomy	2 (1.0)
Pars plana vitrectomy	11 (5.4)
Phacoemulsification	4 (2.0)
DSAEK	1 (0.1)
PKP	1 (0.5)
Tube revision with patch graft	2 (1.0)
Total number of eyes with postoperative interventions^b^	44 (21.6)

*DSAEK* Descemet’s stripping automated endothelial keratoplasty, *PKP* penetrating keratoplasty, *VEGF* vascular endothelial growth factor.

^a^Data are presented as the number of eyes (percentage).

^b^Some patients had more than one intervention.

The visual acuity (logMAR) deteriorated significantly from 0.89 ± 0.83 at the preoperative visit to 1.30 ± 1.09 at the final follow-up visit (P < 0.01). The final visual acuity deteriorated in 118 eyes (57.9%), including eight eyes (3.9%) leading to the no light perception. The final visual acuity improved in 48 eyes (23.5%) and remained in 38 eyes (18.6%).

## Discussion

Some previous studies evaluated the outcome of BGI surgery. Krishna et al. retrospectively evaluated the intermediate-term outcomes of BGI (350-mm^2^ endplate) surgery for 65 eyes^7^. Seah et al. retrospectively assessed the intermediate-term outcomes of BGI (250-mm^2^ and 350-mm^2^ endplates) surgery for 124 eyes of Asian patients^8^. The TVT study prospectively evaluated the 5-year efficacy of BGI surgery for 107 eyes, excluding eyes with NVG^2^. Christakis et al. prospectively assessed the 5-year efficacy of BGI surgery for 247 eyes^5^. Moreover, Matsushita et al. retrospectively analyzed the 3-year outcome of BGI (350-mm^2^ endplate) surgery for 27 eyes of Japanese patients^9^. Other studies with small sample sizes reported the surgical results of BGI separately for each type of glaucoma^10–15^. The present study is unique because it examined 204 eyes of Japanese patients with different types of glaucoma and evaluated the long-term surgical outcome of BGI (350-mm^2^ endplate) surgery.

In the present study, the survival rate at 5 years postoperatively was 72.4% 49.7%, and 24.4% based on criteria A (IOP ≤ 21 mmHg), B (IOP ≤ 17 mmHg), and C (IOP ≤ 14 mmHg), respectively. Matsushita et al. showed that the success rates at 3 years postoperatively were 77.8% (IOP ≤ 21 mmHg) and 48.2% (IOP ≤ 16 mmHg)^9^. Our findings were almost consistent with the results of this previous study from Japan, although the IOP criteria and observation period were slightly different. In the TVT study, the success rates at 5 years postoperatively were 70.2%, 68.2%, and 47.7% based on IOP ≤ 21, ≤ 17, and ≤ 14 mmHg, respectively^2^. In addition, Christakis et al. reported that survival rates at 5 years postoperatively were 65%, 63%, and 52% according to IOP ≤ 21, ≤ 18, and ≤ 15 mmHg, respectively^5^. Compared with these previous reports, the present study had a lower success rate for criteria of IOP ≤ 17 and ≤ 14 mmHg. These inconsistent results might be due to differences in patient background, particularly the type of glaucoma and previous intraocular surgery (filtration surgery, vitrectomy, cataract surgery).

The multivariable analysis in our study identified three risk factors (i.e., age, preoperative IOP, and number of previous intraocular surgery or previous filtration surgery) for surgical failure (Table 3, Supplement Table 1S). Higher preoperative IOP values were associated with surgical failure for criteria B and C. The association between high preoperative IOP and surgical failure of BGI was previously reported^7,15^. This result suggests that in refractory glaucoma with high IOP, for which BGI surgery is indicated, it is difficult to reduce the IOP ≤ 17 mmHg. Investigations showed that previous intraocular surgeries are associated with poor surgical prognosis^7–9,15,16^. Similarly, the present study identified a higher number of previous intraocular surgeries as the risk factor of surgical failure for criterion B and C. Välimäki et al. showed that non-functioning blebs expressed more extracellular matrix components and activated fibroblasts than the functioning blebs in eyes that had undergone tube-shunt surgery^17^. Furthermore, intraoperative conjunctival incisions cause fibroblast activation in the subconjunctival tissue^18^. Therefore, repeated ocular surgery including filtration surgery may cause hypoplasia of the bleb following BGI surgery^16^. It might be difficult to control an IOP ≤ 17 mmHg in eyes that have been subjected to prior intraocular surgeries. Moreover, younger age was linked to an increased risk of surgical failure for criterion B and C. Previous reports also suggested that younger age was a risk factor for failure of BGI surgery^7,8,13^. As previously mentioned, the wound healing process by active fibroblasts may be associated with surgical failure of BGI. Fujiwara et al. reported that age-related impairments in wound healing were associated with fibroblast dysfunction^19^. Collectively, the available evidence indicates that aging may impair wound healing and increase the surgical success rate of BGI.

The present study demonstrated that the mean IOP decreased significantly from 32.7 ± 9.7 mmHg preoperatively to 13.1 ± 3.9 mmHg at 5 years after surgery, and the mean number of glaucoma medications decreased from 3.7 ± 1.2 to 1.8 ± 1.9. Previous studies showed that the postoperative IOP and number of glaucoma medications after BGI surgery were 13–15 mmHg and 1–2, respectively^2,5,9^. Our analysis on the efficacy of decreases in IOP and the number of glaucoma medications yielded consistent findings with those reports.

In the present study, the early postoperative complication rate was 40.2%; this rate was higher than that reported in previous studies^9,20^. Hyphema, choroidal detachment, vitreous hemorrhage and flat anterior chamber were the most common complications noted in our study. The frequency of hyphema was more frequent than previous reports^9,20^. Few cases of hyphema occurred in a study that did not include NVG or include fewer NVG^9,20^. Hyphema is a common complication after filtration surgery in neovascularization of the anterior chamber, particularly in NVG^21^. The frequency of vitreous hemorrhage was higher than previous study without NVG and pars plana insertion of tube^9^. Tube insertion at the pars plana penetrates the ciliary body, which is rich in blood vessels, and poses a risk of vitreous hemorrhage. The frequency of choroidal detachment and flat anterior chamber was similar to previous reports^9,20^. Therefore, differences in the frequency of hyphema and vitreous hemorrhage may have caused differences in the rates of complications between our study and previous investigations.

In this study, the late postoperative complication rate was 13.7%; this rate was lower than that reported in previous studies^9,20^. Persistent corneal edema (bullous keratopathy) occurred more frequently in a study that included only anterior chamber insertion of tube^20^. Tube insertion in the anterior chamber causes a greater loss of corneal endothelial cell density than insertion in the ciliary sulcus or pars plana^22–26^. Our study included tube insertion in the ciliary sulcus or pars plana (40.7%); consequently, persistent corneal edema may have occurred less frequently in this analysis. Another study included additional glaucoma surgery and loss of light perception as late postoperative complications^9^. After adjusting for this difference, the complication rates appeared almost identical.

In the present study, we detected bullous keratopathy in 10 eyes; of those, seven were cases of exfoliation glaucoma and tube insertion in the anterior chamber. We previously reported that tube insertion in the anterior chamber and exfoliation enhance the corneal endothelial cell loss^22^. Thus, we should select the tube insertion position more carefully when performing tube-shunt surgery for exfoliation glaucoma. We think that the tube should be inserted into the ciliary sulcus or pars plana to protect the corneal endothelial cell, particularly in exfoliation glaucoma. For eyes that have undergone previous vitrectomy, we select the pars plana for the tube insertion position. In our study, endophthalmitis occurred in one eye in which the endplate was placed at the inferior quadrant. Following trabeculectomy, the presence of a filtering bleb at the inferior versus the superior quadrant has been associated with an increased risk of bleb-related endophthalmitis^27,28^. Placement of the endplate of tube-shunt surgery in the inferior quadrant has been associated with tube erosion, which is a major cause of endophthalmitis^29^. Indeed, our case of endophthalmitis developed the tube erosion and resulted in the infection. Therefore, if possible, surgeons should place the endplate in the superior quadrant to reduce the risk of infection after BGI surgery.

We performed postoperative interventions in 44 eyes (21.6%). The frequency of postoperative interventions was similar to that reported in a previous study^20^. Anterior chamber reformation was the most common postoperative intervention in our study (7.8%); however, this complication occurred more frequently in this investigation than in the previous study^20^. In the current study, we did not use the rip cord in the tube; the tube was released all at once instead of in stages, resulting in a rapid decrease in IOP. Therefore, there might have been many cases of flat anterior chamber that required reformation.

This study has some limitations owing to its retrospective nature. Firstly, the method of postoperative follow-up was not standardized. The use of glaucoma medication postoperatively and during the follow-up period was at the discretion of the surgeon. These differences in postoperative management by the surgeon may have affected the success rates. Secondly, we were unable to collect some clinical data regarding the optic nerve, visual field, and corneal endothelial cell density. These data are important for evaluating the efficacy and complications of BGI surgery. Hence, additional prospective studies are warranted to address these limitations.

In conclusion, BGI surgery achieved significant long-term decreases in IOP and the number of glaucoma medications. BGI surgery may be effective for refractory glaucoma in Japanese patients. Nevertheless, postoperative interventions due to complications are required in a relatively large number of cases. We should improve the surgical method of BGI to prevent the development of serious complications.

### Supplementary Information

Supplementary Table 1.

## Data Availability

Data is fully available upon reasonable request to corresponding author.
