# Supplementary material for: Long-term outcomes of Baerveldt glaucoma implant surgery in Japanese patients
Source: Sci Rep. 2023 Aug 31;13:14312. doi: 10.1038/s41598-023-41673-6 (PMC10471744; doi:10.1038/s41598-023-41673-6)
Supplement: Supplementary file 1 — Supplementary Table 1. [file 41598_2023_41673_MOESM1_ESM.docx]

**Supplement Table 1S.** Multivariable analysis to identify prognostic risk factors for failure using Cox proportional hazards regression models.

|  |  | | Criterion | |  | |
| --- | --- | --- | --- | --- | --- | --- |
|  | A | | B | | C | |
|  | HR (95% CI) | P-value | HR (95% CI) | P- value | HR (95% CI) | P- value |
| Age per year  Type of glaucoma (NVG/other)  Preoperative IOP per mmHg  Preoperative glaucoma medication per each  Combined surgery (alone/combined cataract surgery)  Previous filtration surgery | 0.99 (0.97–1.01)  1.13 (0.57–2.14)  1.02 (0.99–1.05)  0.97 (0.76–1.25)  1.09 (0.49–2.89)  1.13 (0.62–2.07) | 0.47  0.72  0.085  0.78  0.85  0.68 | 0.98 (0.96–0.99)  1.17 (0.70–1.92)  1.03 (1.00–1.05)  1.01 (0.84–1.22)  1.16 (0.64–2.26)  1.61 (1.01–2.56) | <0.01  0.54  0.024  0.94  0.64  0.045 | 0.98 (0.97–0.99)  1.15 (0.75–1.73)  1.02 (1.00–1.04)  1.03 (0.90–1.18)  1.09 (0.68–1.84)  1.49 (1.03–2.15) | 0.022  0.52  0.016  0.72  0.74  0.034 |

Abbreviations: CI, confidence interval; HR, hazard ratio; IOP, intraocular pressure; NVG, neovascular glaucoma.
